# Neglecting the neglected during the COVID-19 pandemic: the case of leptospirosis in Sri Lanka

**DOI:** 10.4178/epih.e2022015

**Published:** 2022-01-10

**Authors:** Janith Warnasekara, Suneth Agampodi

**Affiliations:** 1 Department of Community Medicine, Faculty of Medicine and Allied Sciences, Rajarata University of Sri Lanka, Anuradhapura, Sri Lanka; 2 Section of Infectious Diseases, Department of Internal Medicine, Yale University School of Medicine, New Haven, CT, USA

**Keywords:** Leptospirosis, COVID-19, Neglecting, Underestimation, Pandemic, Sri Lanka

## Abstract

The coronavirus disease 2019 (COVID-19) pandemic has disrupted global health systems and affected the transmission dynamics as well as the surveillance of other infectious diseases. This study described the probable effect of the COVID-19 pandemic on the surveillance and control of leptospirosis in Sri Lanka. With 8,579 reported cases and more than 800 estimated deaths, the Sri Lankan public health surveillance system documented the largest outbreak of leptospirosis in Sri Lankan history in 2020. This was the worst infectious disease outbreak Sri Lanka experienced in 2020, but it was neglected, primarily due to the COVID-19 pandemic.

The coronavirus disease 2019 (COVID-19) pandemic has disrupted global health systems, with more than 4.7 million lives already lost and over 234 million cases by the end of September 2021. The excess death rate due to the pandemic may exceed its large direct death toll by more than 50%. In most places, the extra death rate is not accounted for, reported, or documented [1]. It is common to overlook outbreaks of other diseases, sometimes even diseases with higher case fatality rates (CFRs), during a global pandemic, and this tendency may have contributed to these excess deaths. The focus of health systems on COVID-19 can lead to underreporting, underdiagnosis, and suboptimal resource allocation for these “unidentified” outbreaks, resulting in higher death rates [2]. We observed this phenomenon in 2020, when the largest reported outbreak of leptospirosis in Sri Lankan history was neither properly investigated nor given proper attention.

Sri Lanka is considered a hot spot for leptospirosis, with an estimated annual incidence of 52.1 per 100,000 population and 730 annual deaths [3]. Leptospirosis was first documented in Sri Lanka in 1953, and the first scientific investigation was published in 1959 [4]. Since then, cases were frequently identified from almost all districts, but with a particularly high incidence in the wet zone of the country [5]. In 2008, the Epidemiology Unit of Sri Lanka reported 7,423 cases of leptospirosis, the highest number of cases notified to the surveillance system until the last year (2020) (Figure 1).

The reported cases of leptospirosis are considered a gross underestimation and could have been described as part of the COVID-19 pandemic based on previously published causes of underestimations [6]. First, underreporting in public, private, and traditional health sectors has been identified as a major reason for underestimation even without the pandemic [7]. The fear, lockdowns, and changes in focus that accompanied the pandemic could have exacerbated this underestimation. Second, the lack of early diagnostics during the acute phase of the disease often causes misdiagnoses of leptospirosis, since many other diseases, such as hantavirus infections, mimic leptospirosis [6]. In response to the COVID-19 pandemic, the diagnostic focus and the resources were primarily directed towards COVID-19, for which reason there were even more missing cases due to a lack of appropriate diagnostics. Third, the available diagnostics are neither perfect nor effective for diagnosing leptospirosis [6]. The few places engaged in molecular diagnosis, which is now considered as the standard diagnostic procedure for leptospirosis, were also transformed into facilities for COVID-19 diagnosis. Finally, diverse species of *Leptospira* causing different clinical and biochemical manifestations often misdirect the diagnosis of leptospirosis [6]. Some of its symptoms resemble those of influenza, and many mild cases of leptospirosis could have been missed during the COVID-19 pandemic due to this reason. Unexpected outbreaks such as COVID-19 can draw physicians’ attention towards the outbreak and could easily lead to misdiagnoses of leptospirosis in a setting where clinical and epidemiological parameters play an important role in the diagnosis [5].

Sri Lanka had its first full-scale national lockdown due to COVID-19 from March to May 2020. Since then, movement restrictions and small-scale lockdowns took place until May 2021, when another surge of COVID-19 cases was detected [8]. The total numbers of reported cases and deaths due to COVID-19 in 2020 in Sri Lanka were 43,289 and 204, respectively, with a CFR of 0.47% [9]. During the same year, the total number of leptospirosis cases notified to the epidemiology unit of Sri Lanka was 8,579, the highest annual number reported in Sri Lanka (Figure 1). Based on previous calculations of disease underestimation, this number could exceed 12,066. With a reported pooled CFR of 7%, the number of deaths due to leptospirosis in 2020 could have been around 845, 4 times higher than the number of deaths due to COVID-19 [3]. However, the exact number of reported deaths due to leptospirosis is not known, as the “Indoor Mortality and Morbidity Report” (the document containing the mortality data) has not been published yet. Furthermore, the notifications only accounted for 52% of the number documented in the hospital system, and we estimate that the actual number of hospitalizations was around 16,000 [3].

We postulate that the reported data were subject to considerable reporting bias due to COVID-19. While all districts previously known to have endemic leptospirosis reported a higher incidence, Colombo and Gampaha reported a very low incidence (Figure 1). These 2 districts have usually reported the highest numbers in the previous 10-15 years, except for a few years. The low incidence could be partially attributed to the prolonged lockdown of these 2 districts, which had the highest number of COVID-19 cases in 2020. However, despite lockdowns, paddy farming activities—the most frequent route of exposure to leptospirosis in Sri Lanka—continued in these areas [10]. In general, the incidence of most infectious diseases tends to be lower during a pandemic due to social distancing. However, since leptospirosis is transmitted through wet soil and contaminated water, social distancing itself may not have had an effect on disease incidence. We believe that the main reason for the higher number of leptospirosis cases even with the lockdown is that paddy farming, which constitutes the main source of leptospirosis infections in Sri Lanka, was allowed to continue [10]. Individuals who previously engaged in part-time farming in addition to full-time employment also had the opportunity to spend more time in paddy fields. While the transmission of other infectious diseases was interrupted, social distancing and the lockdown could have increased exposure to leptospirosis.

Unlike the 2008 outbreak of leptospirosis, where a substantial amount of disease investigation took place, the 2020 outbreak heretofore went unnoticed by the healthcare system and researchers. The total number of COVID-19 cases reported in 2020 was 43,289, which is negligible compared to the surge of cases after April 2021 [8]. The current pandemic has almost disabled the routine healthcare system, with more than 2,500-3,000 new cases each day. The effect of this additional caseload on the diagnosis and management of other diseases could be substantial, as we have seen with leptospirosis in 2020. Despite already being neglected, the death toll due to common neglected tropical diseases such as leptospirosis could be unprecedently high if health systems are not vigilant and maintain surveillance and response for other diseases as well.

This study has several limitations. A comprehensive overview of excess deaths due to leptospirosis could be obtained by comparing the CFR from 2020 with those of previous years. However, mortality data on leptospirosis have yet to be made available from the Ministry of Health, Sri Lanka.

## Figures and Tables

**Figure 1.**
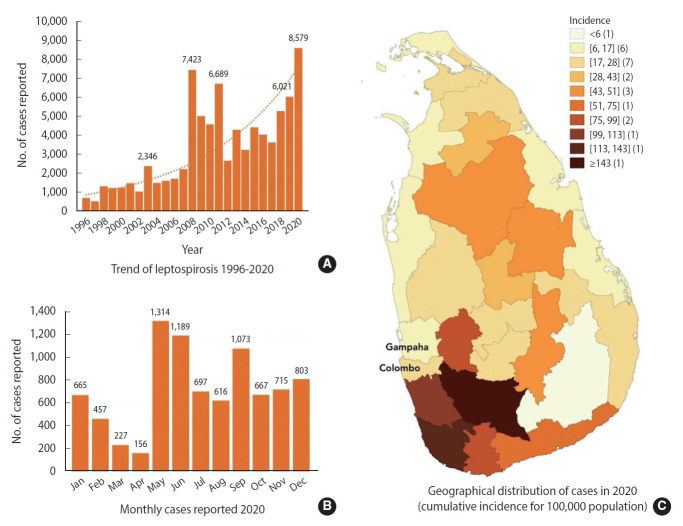
Trend of leptospirosis from 1996 to 2020 (A), monthly leptospirosis cases in 2020 (B), and spatial distribution of leptospirosis (C) in Sri Lanka.
